# 

**Published:** 1860-08

**Authors:** 


Vol. I.] AUGUST, 1860. [No. 8.

### THE CHICAGO
### MEDICAL EXAMINER

A Monthly Journal, devoted to the Educational, Scientific, and practical interests of the Medical Profession.

EDITED BY

N. S DAVIS, M. D.

Professor of Principles and Practice of Medicine and Clinical Medicine, in the Medical Department of Lind University.

AND

E. A. STEELE, M. D.

Terms—$2.00 per annum, in advance.

CHICAGO:

Wm. Cravens & Co. Printers and Publishers,

No. 132 LAKE STREET.

